# How *social* is social media for transgender and gender-diverse youth? Association of online social experiences with internalizing mental health problems

**DOI:** 10.1007/s00787-024-02396-9

**Published:** 2024-03-17

**Authors:** Lena Herrmann, Claus Barkmann, Carola Bindt, Sarah Hohmann, Saskia Fahrenkrug, Inga Becker-Hebly

**Affiliations:** Department of Child and Adolescent Psychiatry, Psychotherapy and Psychosomatics, University Medical Centre Hamburg-Eppendorf, Hamburg, Germany

**Keywords:** Transgender, Gender-diverse, Social media, Internet, Mental health

## Abstract

Adolescents spend a critical amount of their free time on the Internet and social media. Transgender and gender-diverse (TGD) adolescents, who report elevated rates of mental health issues, especially internalizing problems, have both positive and negative online social experiences (e.g., support and cyberbullying). This can have both beneficial and/or harmful effects on their mental health. Given the lack of research, the present study examined TGD adolescents’ online (social) experiences and the association of positive and negative online social experiences with internalizing problems. The sample consisted of *n* = 165 TGD adolescents (11–18 years) diagnosed with gender dysphoria who attended a Gender Identity Service for children and adolescents (Hamburg GIS) in Germany between January 2020 and December 2022 during the COVID-19 pandemic. Positive (use of online support networks) and negative online social experiences (cyberbullying or other adverse online interactions) were assessed using study-specific items and internalizing problems using the Youth Self-Report. Frequencies of various online (social) experiences were analyzed, and a multiple linear regression analysis was performed to test their association with internalizing problems. In total, 42% of participants reported positive online social experiences (use of online support networks) and 51% of participants reported negative online social experiences (cyberbullying or other adverse online interactions). There was no significant association between negative online social experiences and internalizing problems but between positive online social experiences and more internalizing problems (adjusted *R*^2^ = .01). TGD adolescents may seek online support, especially when struggling with mental health problems. Therefore, it is crucial to support youth navigating these online spaces more safely and positively and to empower them to buffer against potentially harmful experiences. Furthermore, strengthening offline relations with peers and family members is pivotal, given their importance for TGD adolescents’ mental health.

## Introduction

The Internet and social media have fundamentally changed social interactions and experiences, especially among adolescents. Transgender and gender-diverse (TGD) adolescents have both positive (e.g., seeking and/or receiving support) and negative experiences (e.g., cyberbullying) online with potentially beneficial versus harmful effects on their mental health [1]. While the impact of offline social experiences (e.g., peer problems or family support) on the mental health of TGD adolescents is well-documented [2, 3], there is little knowledge about how various online social experiences are associated with psychological problems in TGD adolescents.

Several studies document that TGD adolescents (i.e., who do not or not entirely identify with their birth-assigned sex) report significantly more behavioral and emotional problems, especially internalizing ones, and elevated rates of depression, suicidality, self-harm, and eating disorders in comparison to their cisgender peers (i.e., who identify with their birth-assigned sex) [3–6]. According to the minority stress model, these mental health disparities can be caused by experiencing stigma, prejudice, and discrimination because of one’s minority status [7, 8]. The effect of these so-called minority stressors can be buffered against by coping mechanisms and social support, for example, by the lesbian, gay, bisexual, transgender, and queer (LGBTQ) community [9]. Consistent with the minority stress model, studies indicate that difficulties in social interactions with peers or so-called poor peer relations have a significant and negative impact on the psychological functioning of young TGD individuals [2, 3, 10, 11]. Additionally, family support and general family functioning (or the lack thereof) appear to contribute to better (or worse) psychological outcomes in TGD children and adolescents [3, 10, 12]. In summary, research has demonstrated the importance of offline social experiences and interactions in affecting the mental health of TGD youth. Similarly, positive versus negative online social experiences could have either positive or negative effects on the mental health of TGD youth.

The Internet and social media have substantially changed social relationships, interactions, and experiences. Social media is a communication format where one can produce and share content, create profiles, interact with others, and build social networks [13, 14]. Nearly all adolescents in the USA use at least one social media platform (95% use YouTube) and as many as 97% are online daily [15]. In Germany, where the present study took place, 88% of adolescents are online daily and spend, on average, 4 h on the Internet [16]. These numbers from the general population are similar to those of a recent German clinical study on TGD adolescents who were, on average, online for 4.2 h daily [17]. Of importance for the present study, which was conducted during the COVID-19 pandemic, is that adolescents from the German general population spent, on average, more time online at the beginning of the COVID-19 pandemic than before and that these numbers decreased in 2021 but were still elevated in comparison to the pre-pandemic levels [16, 18, 19].

In meta-analyses, adolescent social media use is associated with both worse mental health outcomes and higher levels of well-being, highlighting the complexity of the association and the need to address other risk and protective factors and to identify for whom social media use has which effect [20]. Among LGBTQ youth specifically, the association between social media use and mental health outcomes may be different and even more complex than among non-LGBTQ youth [21], given the multiple benefits (e.g., easy and anonymous access to identity-related information) and risks (e.g., being exposed to homophobic and transphobic content) of the Internet and social media for LGBTQ youth [22]. While most studies are drawn from broader LGBTQ samples (which are, therefore, also described in the following summary), there is a need to recognize and examine the unique experiences of TGD youth that may differ from other groups under the LGBTQ umbrella. For instance, in a recent study examining a clinical sample similar to the present one, 60% of TGD adolescents experimented with their gender identity online, about 30% came out online first, and 90% had socially transitioned online. About half of TGD adolescents each had sought online support for LGBTQ or TGD people and reported negative online social experiences [17].

Social support through connecting online to other like-minded peers and the LGBTQ community can help minority youth feel less alone, isolated, or depressed [22–24]. LGBTQ youth may also feel safer and more supported participating in online than in offline LGBTQ communities [25]. In a recent qualitative study, socially isolated LGBTQ youth living in rural areas of the USA frequently sought support in online communities and groups when they were not feeling emotionally well or had problems. By connecting to like-minded peers in online groups, they described feeling a sense of belonging [26]. For TGD youth, it may be especially difficult to find like-minded peers and other TGD youth offline (because of their minority status). Thus, meeting other TGD individuals online can be an important source of support and make them feel less lonely [27]. In an Australian community-based study called “Trans Pathways,” three-quarters of TGD youth used social media to help themselves feel better, for example, by meeting other TGD people online who told them how life could be better [28]. The Internet and social media can also be a source of support when dealing with specific mental health problems. For instance, in an Australian quantitative study conducted during pandemic-related social isolation, 82% of TGD youth had used social media to seek support for suicidal thoughts or self-harm [29].

However, the Internet and social media can also bear risks and negative experiences. One potentially harmful experience is cyberbullying. Cyberbullying can be defined as communicating aggression or causing harm to others with the help of digital media, for example, by spreading rumors or threatening messages online [30]. Compared to their heterosexual and cisgender counterparts, LGBTQ youth are significantly more likely to experience cyberbullying, with 25 to 40% of LGBTQ youth and up to 50% of TGD youth reporting cyberbullying [17, 27, 31, 32].

Cyberbullying and other negative online social experiences contribute to the psychological distress of LGBTQ youth and young adults and are associated with higher levels of suicidality and depression [30, 31, 33, 34]. Compared to offline bullying, perpetrators may feel more confident online because of the anonymity, making cyberbullying and transphobic behavior more likely and “easy” to carry out [24, 35]. As online information is easy to access and share and because of its’ persistence, adolescents seem to perceive cyberbullying as worse than offline bullying [33, 36]. However, when comparing the effect of both forms on LGBTQ youths’ mental health problems, cyberbullying seems to have a similarly large effect as offline bullying. Furthermore, experiencing both forms of victimization at the same time might increase the risk of depression and suicidality even more [30, 31, 33].

## Current study

Although there are several, mainly US American, studies on the associations of positive online social experiences (use of online support networks) and negative online social experiences (cyberbullying or other adverse online interactions) with especially internalizing problems in LGBTQ youth, the experiences of TGD youth are not assessed or not differentiated from those of sexual minority youth in most studies. Thus, quantitative studies focusing on these associations in TGD youth specifically and studies from Europe are currently lacking. Therefore, the present study focused on several specific (social) experiences TGD adolescents may have on the Internet and social media and how these are associated with internalizing problems. We aimed to answer the following research questions:

1. Which experiences (experimenting with and expressing gender identity online, positive vs. negative online social experiences) do TGD adolescents have on the Internet and social media?
2. How are positive (use of online support networks) vs. negative online social experiences (cyberbullying or other adverse online interactions) associated with internalizing problems in TGD adolescents? Is there an interaction effect?

We hypothesized that positive online social experiences (use of online support networks) would be associated with fewer and negative online social experiences (cyberbullying or other adverse online interactions) with more internalizing problems in TGD adolescents.

## Methods

### Study design

Data came from a cross-sectional study assessing a clinical cohort of TGD adolescents with psychometric self-report questionnaires. The data collection took place at the Hamburg Gender Identity Service for children and adolescents (Hamburg GIS) between January 2020 and December 2022. Thus, the study period fell within the time of the COVID-19 pandemic. The Hamburg GIS at the University Medical Center Hamburg-Eppendorf in Germany offers specialized diagnostics, counseling, and gender-affirming treatment to TGD youth and to youth who have questions about their gender or sexual identity. All families attending the Hamburg GIS are invited to participate in the study at their first appointment, thus before undergoing any form of counseling or treatment.

As part of a research project on “Gender- and Neurodiversity in Childhood and Adolescence” ongoing since 2020, the present study evaluated various updated questionnaires on the psychological health and the life experiences of TGD youth. The local ethics committee approved the study (12/2019-PTK-HH). Participation is voluntary, i.e., counseling or treatment is offered regardless of participation or nonparticipation in the study. All participants completed an informed consent form for their voluntary participation.

### Participants

**Fig. 1:**
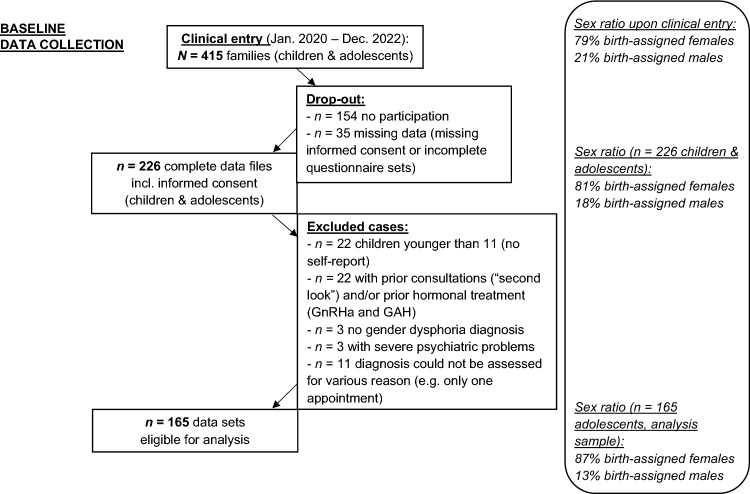
Participants and sex ratios at the Hamburg GIS for children and adolescents

The wider study population included children (aged 5–10 years) and adolescents (aged 11 years and above) who attended the clinic between January 2020 and December 2022. In this period, 415 families had presented to the Hamburg GIS (79% assigned female at birth [AFAB], 21% assigned male at birth [AMAB]; Fig. 1). Incomplete datasets, children, youth without a diagnosis of gender dysphoria, and other cases (for various reasons) were excluded from the analyses (see Fig. 1). The final sample comprised 165 TGD adolescents aged 11 to 18 years (87% AFAB, 13% AMAB) with a clinical diagnosis of gender dysphoria.

### Variables and instruments

#### Sociodemographic as well as psychosocial characteristics and time spent online

The following sociodemographic characteristics were evaluated: birth-assigned sex, age at assessment (upon clinical entry), current gender identity, citizenship, parental socioeconomic status, and parental marital status and living situation. For detailed descriptions of the sociodemographic variables, see Levitan et al. [3] and Herrmann et al. [17].

In addition, we evaluated the following psychosocial characteristics to control for two predominately offline social experiences, which are well-documented risk and protective factors for internalizing problems in TGD youth (see Introduction): general family functioning and poor peer relations. The McMaster Family Assessment Device (FAD) was used to assess general family functioning [37]. The FAD has been used in previous studies on TGD youth [3, 10, 38]. For the present study, we used only the FAD subscale on general family functioning, which consists of 12 items, such as feeling accepted and understood (e.g., “Individuals are accepted for what they are”), supporting each other (“In time of crisis we can turn to each other for support”) and expressing feelings to each other (“We can express feelings to each other”). The adolescents rate the items on a 4-point scale (from 1 = “strongly agree” to 4 = “strongly disagree”). An average general family functioning score is created by adding the items and dividing the sum by the total number of items, resulting in a range from 1 to 4, with higher scores indicating lower levels of family functioning. For categorical analyses (problematic or unhealthy family functioning), the cutoff is 2.17 [39]. The internal consistency of the scale was good in the present study (Cronbach’s $\alpha$ = .89).

Poor peer relations were assessed with the German 1991 version of the Youth Self-Report (YSR) [40, 41]. The YSR includes 119 items that adolescents rate on a 3-point scale ranging from 0 (“not true”) to 2 (“very true or often true”) concerning the past six months. An index for poor peer relations was created based on the following items: Item 25 (“I don’t get along with other kids”), Item 38 (“I get teased a lot”), and Item 48 (“I am not liked by other kids”). The poor peer relation index has been used in several clinical studies to assess problematic social interactions of TGD children and adolescents with peers [2, 3, 10]. The index can range from 0 to 6. Higher scores reflect poorer peer relations. In the present study, the internal consistency of the index was acceptable to questionable (Cronbach’s $\alpha$ = .66).

To control for the quantity of Internet use, time spent online was measured with an item of the Trans Youth Social Media Questionnaire (TYSMQ). The TYSMQ is a self-constructed self-report questionnaire on TGD adolescents’ online activities and experiences. For its construction, we used and adapted items from two representative German studies on adolescents’ free time and media activities [19, 32]. We also added items to reflect the unique experiences of TGD adolescents. The questionnaire has already been used in another clinical study on TGD adolescents [17]. For time spent online, adolescents rated an item (“How many hours do you spend online/on the Internet daily?”) on an 8-point scale ranging from “none” to “7 or more hours.” Later, answers were dichotomized (0 = up to 5 h daily and 1 = more than 5 h daily) for analysis purposes.

#### Online (social) experiences

Various online (social) experiences were measured with the TYSMQ and with single items. The following online (social) experiences were evaluated in the present study: experimenting with gender identity online (“Did you experiment with your gender identity on the Internet/social media before you did in everyday life?”*)*, coming out online first (“Did you come out on the Internet/social media before you told your friends/parents?”), gender role online (“In which role or gender do you live on the Internet/social media?”), feeling understood and accepted in different life domains (e.g., “Do you feel understood and accepted on the Internet/social media for who you are?”), positive online social experiences (use of online support networks), and negative online social experiences (cyberbullying or other adverse online interactions). For more details on these items and the TYSMQ, please refer to Herrmann et al. [17].

For positive online social experiences (use of online support networks), adolescents were asked: “Do you visit online (Facebook) groups/forums or platforms that focus on networking, support, or treatment of transgender or LGBTQ people?” The item could be rated on a 4-point scale ranging from 0 (“never”) to 3 (“yes, often”). For the analysis, answers were divided into two categories: 0 = no (never) and 1 = yes (rarely too often).

For negative online social experiences (cyberbullying or other adverse online interactions), adolescents were asked whether they had ever had negative experiences online or experienced cyberbullying online or on social media. The item was rated on a 4-point scale from 0 (“never”) to 3 (“yes, often”). Similar to the positive online social experiences, two categories for negative online social experiences (0 = no and 1 = yes) were built. In addition, adolescents were able to indicate whether the negative online social experiences had been related to their gender identity or sexual orientation and whether they had been victim, perpetrator, or both victim and perpetrator of this behavior.

#### Internalizing problems

Internalizing problems were assessed with the YSR [40, 41]. Using the German population-based, age-specific, and sex-specific norm scores by Döpfner et al. [40], *T* scores for the three YSR scales (total problem score, internalizing, and externalizing problems) were computed to determine whether the scores of the present study were within the normal range of the German population. Furthermore, clinical range scores (> 90th percentile;* T* score > 63) were calculated. In the present study, the internal consistency of the internalizing scale was excellent (Cronbach’s $\alpha$ = .92).

For exploratory purposes and to evaluate psychological functioning more comprehensively, YSR scores for externalizing and total problems (sum of all problems) were additionally calculated, and an index for suicidality was created. As suggested by the YSR manual [40] and to avoid artificial conflation, we excluded the following items for the calculation of the total problem score: asthma (Item 2), allergies (Item 4), socially desirable items (16 items), and cross-gender identification (Item 5 and Item 110). As described in previous studies [2, 42], Items 84 and 85 were set to zero if the free-text answers were gender-related. The internal consistencies of the externalizing scale (Cronbach’s $\alpha$ = .86) and the total problem scale (Cronbach’s $\alpha$ = .95) were good to excellent. In addition, two items from the YSR were used to create an index for suicidality, as in other clinical studies on TGD adolescents [4, 6, 43]: Item 18 on self-harming behavior and suicide attempt (“I deliberately try to hurt or kill myself”) and Item 91 on suicidal ideation (“I think about killing myself”). The sum score of the index ranges from 0 to 4, with higher scores indicating higher levels of suicidality. In the present study, the internal consistency of the index was acceptable to questionable (Cronbach’s $\alpha$ = .67).

### Statistical analyses

*T*-tests and chi-square tests (or Fisher’s exact tests) were conducted to explore possible sex differences (AFAB vs. AMAB) in the sociodemographic and psychosocial characteristics and the time spent online and various online (social) experiences. Paired *t*-tests were performed to compare the degree of feeling understood and accepted online with different offline life domains. Standardized effect sizes (*d* and odds ratios, *OR*) were calculated to quantify the magnitude of the effect.

For a descriptive evaluation of internalizing problems, the raw scores, *T* scores, and clinical ranges (> 90th percentile; *T* scores > 63) for the YSR internalizing scale were used. In addition, we calculated 95% confidence intervals to compare the present sample with age- and sex-equivalent population-based German norms [40]. A significant deviation from the reference group can be assumed if the confidence intervals are not within the range of the *T* distribution (*M* = 50, *SD* = 10). Vice versa, whenever confidence intervals overlap, there is no significant difference [44]. For exploratory purposes, the externalizing scale, the total problem score, and the suicidality index were evaluated similarly.

For testing our hypotheses, a multiple linear regression analysis was performed. The raw scores of the YSR internalizing scale were used as an outcome variable. Predictors were entered in a block-wise manner. In the first step, the birth-assigned sex, age, general family functioning, and poor peer relations were entered as control variables. In the second step, positive online social experiences (use of online support networks) were added. In the third step, negative online social experiences (cyberbullying or other adverse online interactions) were introduced to the regression analysis. In the fourth step, the interaction between positive and negative online social experiences was added. An a priori power analysis (using G*Power) demonstrated that in a multiple linear regression analysis with 165 cases and seven predictors, a medium effect (*f* = 0.15) can be tested with a power of 95%. For exploratory purposes, similar analyses were conducted with externalizing problems, the total problem score, and the suicidality index as outcomes. As in other similar studies [3, 10], three items on poor peer relations (Items 25, 38, and 48) were excluded from the total problem score when exploring its association with online social experiences because the poor peer relation index was already a predictor in the model.

Single missing values were imputed with the expectation–maximization algorithm [45] and the mean values. SPSS 27 was used for all statistical analyses.

## Results

### Sociodemographic as well as psychosocial characteristics and time spent online

See Table 1 for all results on sociodemographic and psychosocial characteristics and time spent online. TGD adolescents were, on average, 15 and a half years old when presenting to the Hamburg GIS. The vast majority of TGD adolescents (82%) identified as binary (e.g., trans man/boy or male) and 18% as nonbinary or were gender questioning at their initial presentation to the Hamburg GIS. Most adolescents were German citizens and had a medium (53%) or high (39%) (parental) socioeconomic background. The (parental) socioeconomic status of AMAB adolescents was significantly higher than that of AFAB adolescents. There were no other sex differences for any characteristics presented in Table 1.

**Table 1:** Sociodemographic and psychosocial characteristics and time spent online among adolescents assigned female vs. assigned male at birth

	AFAB		AMAB		Total		Group comparisons			
	(*n* = 143)		(*n* = 22)		(*n* = 165)					
	*n*	%	*n*	%	*n*	%	*Χ*^*2*^/FT	*df*	*p*	*OR*
**Current gender identity**										
Binary	116	81.1	20	90.9	136	82.4				
Nonbinary or gender questioning	27	18.9	2	9.1	29	17.6	–	–	.372	0.43
**Citizenship**										
German	140	97.9	21	95.5	161	97.6				
Other	3	2.1	1	4.5	4	2.4	–	–	.439	2.22
**Parents’ marital status and living situation**										
Both parents living together/married	67	46.9	13	59.1	80	48.5				
Other	76	53.1	9	40.9	85	51.5	1.14	1	.285	0.61
**Time spent online**										
Up to 5 h daily	98	68.5	14	63.6	112	67.9				
More than 5 h daily	45	31.5	8	36.4	53	32.1	0.21	1	.647	1.24

	*M*	*SD*	*M*	*SD*	*M*	*SD*	*t*	*df*	*p*	*d*
**Age at assessment** (in years)	15.43	1.34	16.02	1.46	15.51	1.37	− 1.90	163	.059	− 0.44
**Parental socioeconomic status** (Winkler Index)	6.75	1.54	7.45	1.44	6.84	1.54	− 2.02	163	.045	− 0.46*
**General family functioning** (FAD)	2.01	0.60	2.01	0.46	2.01	0.58	− 0.02	163	.988	− 0.00
**Poor peer relations** (YSR)	1.55	1.43	2.00	1.72	1.61	1.47	− 1.33	163	.185	− 0.31

^*^p < .05, *AFAB/AMAB* assigned female/male at birth, *FAD* McMaster Family Assessment Device, *FT* Fisher’s exact test, *YSR* Youth Self-Report

In about half of the cases, both parents lived together or were married. The reported family interactions (general family functioning) were, on average, unproblematic (below the cutoff). In total, 41% of adolescents described problematic family functioning (above the cutoff), and 72% had encountered at least one peer-related problem in the past six months. Most adolescents reported spending up to 5 h on the Internet each day, whereas one-third reported spending more than 5 h online daily.

### Online (social) experiences

See Table 2 for all results on online (social) experiences. Two-thirds of TGD adolescents had experimented with their gender identity online first (then offline), and one-third had come out online first before telling their friends or parents. Additionally, 89% presented themselves not as their birth-assigned sex online but in another gender (role). On the Internet and social media, TGD adolescents felt, on average, rather understood and accepted for who they are. TGD adolescents felt significantly more understood and accepted online than by their parents, classmates/peers, and teachers but significantly less understood and accepted online than by their friends. AMAB adolescents reported to feel significantly less understood and accepted by their peers than AFAB adolescents (*t*(163) = 2.09, *p* = 0.038, *d* = 0.48). There were no other sex differences for any of these variables in our study, meaning that AFAB and AMAB adolescents reported similar online (social) experiences in the present study.

**Table 2:** Online (social) experiences of adolescents assigned female vs. assigned male at birth

	AFAB		AMAB		Total		Group comparisons			
	(*n* = 143)		(*n* = 22)		(*n* = 165)					
	*n*	%	*n*	%	*n*	%	*Χ*^*2*^/FT	*df*	*p*	*OR*
**Experimenting with gender identity online**										
No, experimented in everyday life first	49	34.3	7	31.8	56	33.9				
Yes, experimented online first	94	65.7	15	68.2	109	66.1	0.05	1	.821	1.12
**Coming out online first**										
No, came out with friends/parents first	96	67.1	13	59.1	109	66.1				
Yes, came out online first	47	32.9	9	40.9	56	33.9	0.55	1	.458	1.41
**Gender role online**										
Gender role of their birth-assigned sex	16	11.2	3	13.6	19	11.5				
Gender role of another gender	127	88.8	19	86.4	146	88.5	–	–	.722	0.80
**Positive online social experiences (use of online support networks)**										
No (never)	86	60.1	9	40.9	95	57.6				
Yes (rarely, occasionally, or often)	57	39.9	13	59.1	70	42.4	2.89	1	.089	2.18
**Negative online social experiences (cyberbullying or other adverse online interactions)**										
No (never)	70	49.0	11	50.0	81	49.1				
Yes (rarely, occasionally, or often)	73	51.0	11	50.0	84	50.9	0.01	1	.927	0.96

	*M*	*SD*	*M*	*SD*	*M*	*SD*	*t*	*df*	*p*	*d*
**Feeling understood and accepted in different life domains**										
Internet/social media	3.34	0.75	3.05	1.13	3.30	0.81	*Comparison to other domains*			
Parents	2.64	1.17	2.77	1.02	2.66	1.15	6.25	164	< .001	0.49***
Friends	3.50	0.75	3.45	0.74	3.49	0.75	− 2.52	164	.013	− 0.20*
Classmates/peers	2.34	1.16	1.77	1.31	2.26	1.19	10.23	164	< .001	0.80***
Teachers	2.66	1.11	2.27	1.42	2.61	1.16	7.03	164	< .001	0.55***

^*^p < .05, ***p < .001, *AFAB/AMAB* assigned female/male at birth, *FT* Fisher’s exact test

Regarding positive online social experiences, 42% of TGD adolescents had used online support networks for TGD or LGBTQ individuals (16% rarely, 14% occasionally, and 12% often). AMAB adolescents tended to use online support networks more often than AFAB adolescents, but the difference was statistically nonsignificant. Negative online social experiences were similarly common: Half of TGD adolescents reported cyberbullying or other adverse online social experiences (31% rarely, 13% rarely, and 7% often). Negative online social experiences were in 67% of the cases related to the TGD adolescents’ gender identity (35%), their sexual orientation (2%), or both (30%). Among TGD adolescents who reported negative online social experiences, the majority (55%) had been the target of cyberbullying; 5%, perpetrators; and 19%, both. Another 21% had neither been victims nor perpetrators of cyberbullying and reported other adverse online interactions instead (e.g., “It happened in groups that I wasn't in myself. Friends told me about it”).

### Internalizing problems

Table 3 shows the results for internalizing problems. Compared to adolescents from the German norm population, TGD adolescents had, on average, significantly higher *T* scores (95% CI not including *M* = 50), which were elevated by almost 2 *SD*. In total, 62% of TGD adolescents scored within the clinical range of internalizing problems (> 90th percentile; *T* scores > 63). There were no significant sex differences (overlapping 95% CIs).

**Table 3:** Internalizing problems according to the birth-assigned sex and compared to German norm scores

	Raw scores			*T* scores (TGD adolescents with reference to the norm)			Clinical range (*T* scores > 63)	
	*M*	*SD*	95% CI	*M*	*SD*	95% CI	*n*	%
**YSR internalizing scale**								
Assigned female at birth	24.31	12.00	[22.32; 26.29]	68.08	12.12	[66.08; 70.09]	86	60.1
Assigned male at birth	22.14	10.08	[17.67; 26.60]	70.18	10.80	[65.39; 74.97]	17	77.3
Total	24.02	11.76	[22.21; 25.83]	68.36	11.94	[66.53; 70.20]	103	62.4

*Note*. Age and birth-assigned sex-equivalent German norm YSR *T* scores with *M* = 50 and *SD* = 10 were derived from Döpfner et al. (1998)

*TGD* transgender and gender diverse, *YSR* Youth Self-Report

### Association of positive vs. negative online social experiences with internalizing problems

For the results on the association of positive vs. negative online social experiences with internalizing problems, see Table 4. In the final model of the multiple linear regression analysis, female birth-assigned sex, lower levels of general family functioning, poorer peer relations, and positive online social experiences (use of online support networks) were significantly associated with reporting more internalizing problems. Neither negative online social experiences (cyberbullying or other adverse online interactions) nor the interaction between positive and negative online social experiences were significant predictors for internalizing problems. The final model explained 44% of the variance in internalizing problems, while all control variables together explained 42%, positive online social experiences (use of online support networks) 1%, negative online social experiences (cyberbullying or other adverse online interactions) 0.2%, and the interaction between positive and negative online social experiences 0.3%.

**Table 4:** Association of positive (use of online support networks) vs. negative online social experiences (cyberbullying or other adverse online interaction) with internalizing problems (YSR raw scores)

	*b*	*SE b*	*ß*	*p*
Intercept	3.44	7.99		.668
Birth-assigned sex (0 = female, 1 = male)	− 4.12*	2.08	− .12	.049
Age in years	− 0.17	0.53	− .02	0.754
General family functioning (FAD)	7.80***	1.28	.38	< .001
Poor peer relations (YSR)	3.38***	0.51	.42	< .001
Positive online social experiences (0 = no, 1 = yes)	4.42*	2.10	.19	.037
Negative online social experiences (0 = no, 1 = yes)	3.38	1.87	.14	.073
Interaction (positive x negative social online experiences)	− 3.95	2.84	− .15	.166

Results of the final model of the multiple linear regression analysis: *F* (7, 157) = 19.10, adjusted *R*^*2*^ = .44, *p* < .001

^*^p < .05, ***p < .001*,* *FAD* McMasters’ Family Assessment Device, *YSR* Youth Self-Report

### Exploratory data analyses

In addition to the hypothesis testing, exploratory analyses were performed (see Table 5, 6, 7, 8). In comparison to internalizing, externalizing problems were less common but still elevated in TGD adolescents (*T* scores 0.6 *SD* above *M* = 50; Table 5). In total, 12% of TGD adolescents scored within the clinical range of externalizing problems. On the total problem score, TGD adolescents scored 1.5 *SD* higher than the norm population, and 53% scored within the clinical range. Concerning suicidality, more than half of TGD adolescents reported that they sometimes (30%) or often (24%) tried to hurt or kill themselves (YSR Item 18). In addition, 43% thought sometimes (33%) or often (10%) about killing themselves (YSR Item 91). There were no significant sex differences for any of the scores.

**Table 5:** Exploratory analyses of externalizing problems, total problem score, and suicidality index according to the birth-assigned sex and compared to German norm scores

	Raw scores			*T* scores (TGD adolescents with reference to the norm)			Clinical range (*T* scores > 63)	
	*M*	*SD*	95% CI	*M*	*SD*	95% CI	*n*	%
**YSR externalizing scale**								
Assigned female at birth	13.07	7.61	[11.81; 14.33]	56.19	8.92	[54.71; 57.66]	19	13.3
Assigned male at birth	11.64	7.01	[8.53; 14.74]	53.50	7.64	[50.11; 56.89]	1	4.5
Total	12.88	7.53	[11.72; 14.04]	55.83	8.79	[54.48; 57.18]	20	12.1
**YSR total problem score**								
Assigned female at birth	59.79	26.21	[55.46; 64.12]	64.99	10.21	[63.31; 66.68]	77	53.8
Assigned male at birth	53.27	20.19	[44.32; 62.22]	63.82	7.77	[60.37; 67.26]	11	50.0
Total	58.92	25.53	[55.00; 62.85]	64.84	9.91	[63.31; 66.36]	88	53.3
**YSR suicidality index**								
Assigned female at birth	1.38	1.30	[1.17; 1.60]	–	–	–	–	–
Assigned male at birth	0.82	1.14	[0.31; 1.32]	–	–	–	–	–
Total	1.31	1.29	[1.11; 1.51]	–	–	–	–	–

In summary, three exploratory multiple linear regression analyses were conducted. First, lower levels of family functioning and negative online social experiences (cyberbullying or other adverse online interactions) were significantly associated with more externalizing problems, whereas positive online social experiences (use of online support networks) and the interaction (positive x negative online social experiences) were not (Table 6). The final model explained 22% of the variance, of which negative online social experiences (cyberbullying or other adverse online interactions) explained 8%.

**Table 6:** Exploratory analysis for the association of positive (use of online support networks) vs. negative online social experiences (cyberbullying or other adverse online interaction) with externalizing problems (YSR raw scores)

	*b*	*SE b*	*ß*	*p*
Intercept	3.18	6.03		.598
Birth-assigned sex (0 = female, 1 = male)	− 1.40	1.57	− .06	.371
Age in years	− 0.09	0.40	− .02	.817
General family functioning (FAD)	4.45***	0.97	.34	< .001
Poor peer relations (YSR)	− 0.29	0.39	− .06	.455
Positive online social experiences (0 = no, 1 = yes)	1.13	1.58	.07	.477
Negative online social experiences (0 = no, 1 = yes)	4.79***	1.41	.32	< .001
Interaction (positive x negative online social experiences)	− 0.31	2.14	− .02	.886

Results of the final model of the multiple linear regression analysis: *F*(7, 157) = 7.52, adjusted *R*^*2*^ = .22, *p* < .001

^***^p < .001*,* *FAD* McMasters’ Family Assessment Device, *YSR* Youth Self-Report

Second, significant predictors for the total problem score were female birth-assigned sex, lower levels of family functioning, poorer peer relations, and negative online social experiences (cyberbullying or other adverse online interactions) (Table 7). Neither positive online social experiences (use of online support networks) nor the interaction (positive x negative online social experiences) were significant predictors. In total, 44% of the variance was explained. Reporting negative online social experiences (cyberbullying or other adverse online interactions) explained 4% of the variance in the total problem score.

**Table 7:** Exploratory analysis for the association of positive (use of online support networks) vs. negative online social experiences (cyberbullying or other adverse online interaction) with the total problem score (YSR raw scores)

	*b*	*SE b*	*ß*	*p*
Intercept	11.49	16.86		.497
Birth-assigned sex (0 = female, 1 = male)	− 10.02*	4.38	− .14	.023
Age in years	− 0.29	1.11	− .02	.796
General family functioning (FAD)	17.50***	2.70	.41	< .001
Poor peer relations (YSR)	5.10***	1.08	.30	< .001
Positive online social experiences (0 = no, 1 = yes)	7.73	4.42	.15	.082
Negative online social experiences (0 = no, 1 = yes)	12.75**	3.95	.26	.002
Interaction (positive x negative online social experiences)	− 6.04	5.99	− .11	.314

Results of the final model of the multiple linear regression analysis: *F*(7, 157) = 19.12, adjusted *R*^*2*^ = .44, *p* < .001

^*^p < .05, **p < .01, ***p < .001*,* *FAD* McMasters’ Family Assessment Device, *YSR* Youth Self-Report

Third, female birth-assigned sex, lower levels of family functioning, and poorer peer relations were significantly associated with a higher suicidality index (Table 8). There were no significant associations between positive or negative online social experiences nor the interaction (positive x negative online social experiences) and the suicidality index. The final model explained 30% of the variance in the suicidality index.

**Table 8:** Exploratory analysis for the association of positive (use of online support networks) vs. negative online social experiences (cyberbullying or other adverse online interaction) with the suicidality index (YSR raw scores)

	*b*	*SE b*	*ß*	*p*
Intercept	0.76	0.98		.438
Birth-assigned sex (0 = female, 1 = male)	− 0.67**	0.25	−.18	.009
Age in years	− 0.10	0.06	− .11	.107
General family functioning (FAD)	0.76***	0.16	.34	< .001
Poor peer relations (YSR)	0.21**	0.06	.24	.001
Positive online social experiences (0 = no, 1 = yes)	0.41	0.26	.16	.109
Negative online social experiences (0 = no, 1 = yes)	0.45	0.23	.18	.050
Interaction (positive x negative online social experiences)	− 0.02	0.35	− .01	.947

Results of the final model of the multiple linear regression analysis: *F*(7, 157) = 11.22, adjusted *R*^*2*^ = .30, *p* < .001

^*^p < .05, **p < .01, ***p < .001*,* *FAD* McMasters’ Family Assessment Device, *YSR* Youth Self-Report

## Discussion

The present study, which took place during the COVID-19 pandemic, aimed to examine the online (social) experiences of TGD adolescents and the associations of positive (use of online support networks) and negative online social experiences (cyberbullying or other adverse online interactions) with internalizing problems.

Many TGD adolescents had used the Internet and social media to experiment with and express their gender identity. Most TGD adolescents had experimented with their gender identity online first, 34% had come out online first, and nearly 90% presented themselves in another gender (role) online. In addition, they felt significantly more understood and accepted online than in most offline domains. These findings are in line with other studies [17, 31] and highlight the advantages of the Internet and social media for the identity development of TGD adolescents.

About 40% of TGD adolescents had visited online groups or platforms focusing on networking, support, or treatment of TGD or LGBTQ people, underscoring the importance of online support networks for TGD youth, probably especially during special circumstances such as the COVID-19 pandemic. Another study from the Hamburg GIS suggests that TGD adolescents primarily use easily accessible social media and smartphone apps such as WhatsApp, Instagram, and Reddit as online support networks [17]. We hypothesized that using online support networks (as opposed to not using them) would be associated with fewer internalizing problems. However, using online support networks was, in the present study, associated with reporting *more* internalizing problems. This result contrasts also previous studies indicating that online support, for example, by connecting to peers or the LGBTQ community, helps TGD youth to feel better, less alone, or less depressed [22, 23, 26, 28]. As a result, the association might be more ambiguous than previously assumed because TGD youth might seek online support, especially when they are already struggling with emotional problems. However, since we only asked TGD adolescents *if* (and how often) they had visited/used online support networks, we do not know how they used these and whether their experiences were always positive or supportive. Thus, more studies are needed that consider the perceived quality of such online social experiences [46].

Half of TGD adolescents reported negative online social experiences including cyberbullying and other adverse online interactions. These numbers resemble other previous studies [17, 27, 32] and emphasize that the Internet and social media have both advantages and disadvantages for TGD youth. In contrast to other broader studies on LGBTQ youth [30, 31, 33, 34] and our second hypothesis, negative online social experiences (cyberbullying or other adverse online interactions) were not associated with more internalizing problems in TGD youth.

However, different results were found in our exploratory analyses: Here, negative online social experiences (cyberbullying or other adverse online interactions) but not positive online social experiences (use of online support networks) were significant predictors for reporting more externalizing problems and emotional and behavioral problems, in general (total problem score). These results underline the possible impact of negative online social experiences (cyberbullying or other adverse online interactions) on TGD youths’ mental health outcomes. However, for suicidal ideation and behavior (YSR suicidality index), which were similarly common as in other studies [4, 6], neither positive nor negative online social experiences were significant predictors. Hence, further research is needed to disentangle the complex associations between positive vs. negative online social experiences and mental health outcomes in TGD youth. For future studies, it would also be interesting if media literacy skills, the level of moderation on online platforms, or belonging to a subgroup within TGD youth (e.g., nonbinary youth) influence the associations.

In summary, the present findings are only partly in line with the minority stress model [7, 8]: For example, in contrast to the proposed “buffering” effect of support, positive online social experiences were not associated with less, but more internalizing problems. However, in line with the minority stress model and previous studies [3, 10, 12], offline social experiences such as family functioning and poor peer relations were among the strongest predictors for the psychological functioning of TGD youth, highlighting their crucial role in the mental health of TGD adolescents once again. Therefore, these results call for further studies to test the applicability of the minority stress model to online contexts and to clarify if online vs. offline social experiences have different meanings for psychological problems in TGD youth.

### Strengths and limitations

Whereas there are many studies on the associations between positive and negative *offline* social experiences (e.g., family functioning and poor peer relations) and the psychological functioning of TGD youth [2, 3, 10], the associations with various online social experiences are still not well documented. Therefore, the present study contributes novel findings to adolescent TGD health research by highlighting an important (online) life domain where young people spend most of their free time.

Along with the research gap, validated questionnaires for assessing online (social) experiences specifically in TGD youth are missing, which is why we used self-constructed and adapted items (for more details, see Herrmann et al. [17]). However, for other variables, such as internalizing problems, common and validated questionnaires were used (e.g., YSR). As internalizing problems were self-reported, just like most other variables (excluding the socioeconomic status and the nationality), these do not represent clinical diagnoses.

Moreover, the study was conducted during the COVID-19 pandemic, which might have impacted our results given that adolescents spent more time online during the pandemic [18]. Therefore, the impact of online social experiences might be different or smaller after the pandemic, when adolescents spend less time online and more time in real life/offline again. Furthermore, with the cross-sectional design of our study, only associations and not causal relationships could be evaluated, calling for longitudinal research. In addition, the generalizability is limited because the present clinical sample is not representative of TGD adolescents in the general population.

Furthermore, we focused on the use of online support networks as positive online social experiences—which might lack specificity as already mentioned (use vs. quality)—and cyberbullying and “other” adverse interactions as negative online social experiences. There are probably more positive and negative online experiences (including social experiences, but not restricted to them) not examined in the study, which could be crucial for TGD adolescents’ mental health, e.g., building and maintaining friendships online, especially for youth who would otherwise feel isolated [22, 23, 26].

### Implications

Since many TGD adolescents, especially those who reported high levels of internalizing problems, used online support networks, interventions that focus on increasing positive online (social) experiences may be helpful: For example, creating TGD-specific platforms with strong community guidelines [34] or providing mental health support or TGD-specific and accurate information in an interactive but professional way may contribute to more positive experiences.

Additionally, interventions that empower TGD adolescents to buffer against negative online social experiences or even prevent these before happening are needed. For example, educational interventions for improving social media account management (e.g., use of privacy settings) and peer-driven educational programs against cyberbullying could be beneficial [30, 34]. Moreover, media literacy programs aimed at parents could improve their understanding of potential risks and dangers their children may face online and support them in teaching healthy media consumption habits.

Sensitizing clinicians working with TGD youth to help them navigate social media and the internet, for example, by providing helpful online resources or educating them about the potential positive and negative effects of the Internet/social media, may additionally be beneficial. At least, clinicians need to be aware of the side effects the Internet may have on their patients’ mental health.

Finally, given the importance of the relationships and interactions with peers and family members for the mental health of TGD youth in this and similar studies [3, 10, 12], one should not lose sight of their offline social experiences.

## Conclusion

In the present study, positive online social experiences (using online support networks) of TGD adolescents were associated with more internalizing problems, but negative online social experiences (cyberbullying or other adverse interactions) were not. We suggest that TGD adolescents who are already struggling with internalizing problems more often seek online support, but further (longitudinal) research is needed to understand the mechanisms and causal relationships better. Given that TGD youth are at risk for lower levels of psychological functioning and that the Internet and social media are unlikely to disappear but possibly become increasingly indispensable, especially for marginalized groups such as TGD youth, interventions that focus on more positive or safer use of the Internet and social media and equip youth to manage the possible risks are critical.

## Data Availability

The data are not publicly available.
